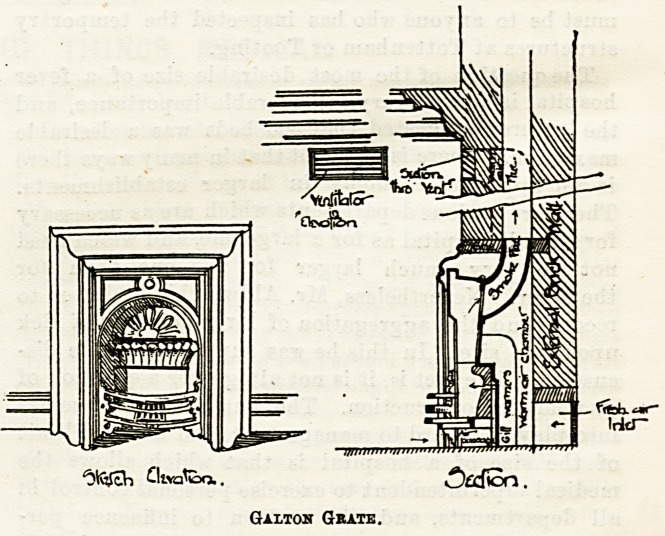# Heating and Ventilation

**Published:** 1895-03-23

**Authors:** 


### PRACTICAL DEPARTMENTS.

### HEATING AND VENTILATION.

The Modern Fireplace.

Since the early years of the present century, and the days of Count Rumford, many and various have been the systems and principles tried by different reformers of the fireplace.

The Sylvester grate was one which found a good deal of favour some fifty years ago, and its main principle is still carried out in some of the best and most modern inventions of to-day. Its chief features were the radiating bars, projecting in a fan-like shape into the room, with the ends in the fire, thus providing a large heated surface, and the introduction of Venetian plates at the back of the grate which could be opened easily with the poker in the place of the usual register door. There was also a hot chamber at the back with openings for the entrance of air from the room and its return. This grate was capable of giving out a good deal of heat, which was open to efficient regulation, but it was superseded in general popularity by others, all of which followed the Rumford principle in the use of fire-brick.

The large adoption of the use of tiles, instead of ironwork,

Teale Grate.

has worked much improvement in the modern grate. Though tiles, being non-conductors of heat, do not radiate much, yet their highly glazed surface reflects considerable warmth, and they have other economical advantages. Their use is the revival of an old custom, and in many a 17th century farmhouse may still be found quaint Dutch-tiled hearths, with the always cosy-looking old-fashioned hob-grate.

Coming down to the present day, probably the fireplace best adapted in most cases to institution as well as to private use is that known as the Pridgin Teale (the Lionel Teale, Fireplace Company, Leeds and London). The wards of the Middlesex Hospital, which are in so many respects models of what wards should be, are fitted throughout with these grates, of which our first illustration shows a specimen. They are as economical in working as they are pleasant to look at, and they give out a splendid heat. Since their adoption at the Middlesex a considerable reduction in the consumption of fuel has been effected. It may be mentioned that large lumps of coal are found to be the most satisfactory in burning. Sir Douglas Galton describes the Teale grate as "a fireplace in a fire-clay receptacle below the level of the hearth, which latter stands about four or six inches above the floor level; this grate is an adaptation of the Sylvester grate in that it has an iron plate carrying the hearth tiles, by means of which they are made hot, and thus the raised

Galton Grate.

hearth of this grate throws out considerable heat and is also useful as a hob."

The accompanying section shows the principle clearly. The ash-pan, if pushed properly home, should not allow the accumulation of any ashes behind, and simply requires daily emptying. In these Middlesex fireplaces it will be seen that the excellent plan has been adopted of having a fixed gas kettle at the side, thus effecting considerable saving of labour.

Among other open fire grates recommended by authorities for hospital and institution use we may mention Dr. Richard Greene's stove, to be seen at the Berrywood Asylum, Northampton, spoken of with especial approval by Mr. Burdett in his "Hospitals and Asylums of the World." The same authority mentions a stove devised by Mr. Saxon Snell, "a combination of two open fires and two coils of hot-water pipes," which has been introduced into some of the large Poor Law infirmaries and has answered satisfactory.

The "Galton" grate, called after its inventor, and originally designed for the War Office, has been largely adopted of recent years in public institutions. By the Local Government Board it has been introduced into many of the most modern Poor Law infirmaries. The wards of the London Temperance Hospital, Hampstead Road, which is noted for the perfection of its internal fittings, are supplied with these grates. The second illustration gives the elevation and section of a specimen of these grates, which are made by Messrs. Yates and Haywood, Upper Thames Street, E.C., by whose permission we give the drawing.

(*To be continued.*)

## Figures and Tables

**Figure f1:**
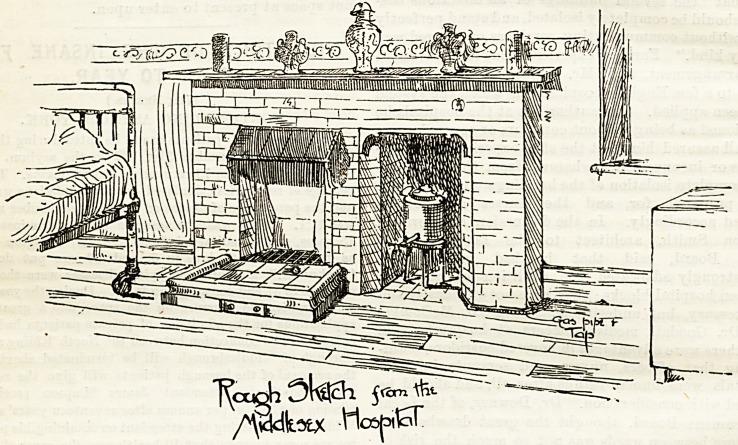


**Figure f2:**
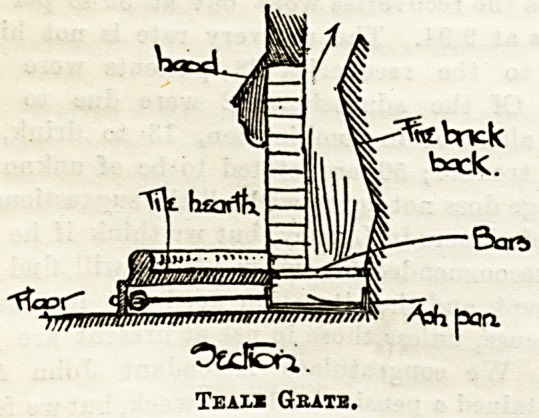


**Figure f3:**